# The Relationship between Individual Personality Traits (Internality-Externality) and Psychological Distress in Employees in Japan

**DOI:** 10.1155/2011/731307

**Published:** 2011-07-26

**Authors:** Masahito Fushimi

**Affiliations:** ^1^Division of Psychiatry, Akita Prefectural Mental Health and Welfare Center, 2-1-51 Nakadori, Akita City, Akita Prefecture 010-0001, Japan; ^2^Division of Psychiatry, Akita Occupational Health Promotion Center, Akita 010-0874, Japan

## Abstract

This study examines the relationship between the internality-externality (I-E) scale as an indicator of coping styles and the Kessler 6 (K6) scale as an indicator of psychological distress and analyzes the effects of sociodemographic and employment-related factors on this relationship. Employees from Akita prefecture in Japan were invited to complete self-administered questionnaires. A uniform pattern of findings emerged in the relationship between the two scales as follows: all the significant correlations were negative, that is, as the I-E score increased, the K6 score decreased. Furthermore, significant effects were observed for the I-E scale regarding sex, age, education, employee type, and employment status and the K6 scale with multiple regression analyses. Among these, the effect of the K6 scale was significant for the I-E scale in both males and females. The results of this study may help improve mental health clinicians' understanding of psychological distress in employees.

## 1. Introduction

The majority of previous literature on stress has studied the relationship between stressors and psychological distress. In addition, several such studies adopted moderators or coping behaviors as factors [1–3]. Several factors serve as potential moderators of stressor-strain relationships; these include Type A behavior pattern, internality-externality (I-E), and hardiness. Type A refers to a behavioral style characterized by ambitiousness, aggressiveness, competitiveness, impatience, potential for hostility, and a hard-driving nature; furthermore, it is characterized by motor responses such as muscle tenseness, a vigorous speech pattern, and rapidity of movement. I-E, which is also called locus of control (LOC), was also adopted as a moderator by several studies [3]. I-E is often described as a personality-like variable that might affect the long-term coping pattern of individuals. LOC refers to the differences in beliefs concerning personal control, represented by the continuum from internality to externality. “Internals” believe that “reinforcements are contingent upon their own behavior, capacities, or attributes.” In contrast, “externals” believe that “reinforcements are not under their personal control but rather are under the control of powerful others, luck, chance, fate, and so forth” [1].

In order to understand the processes related to occupational stress, it is necessary to explore how individuals behave in response to perceived stress (i.e., coping behavior) and to examine the relationship between potentially stressful incidents and psychological distress. Coping behavior is important; however, in analyzing coping with stress, just as they need to examine coping with each stressful incident, there is also a need to explore the “coping style” [2, 3]. Coping style refers to any long-term pattern of coping behavior exhibited by an individual, resulting either from how the individual tends to appraise events or from semihabitual behavior that s/he employs. Not all coping takes place only during stressful incidents or episodes. It is important to study long-term coping styles because psychological distress builds up over months or years, rather than as a mere response to a single stressful incident. Consequently, this study does not focus on individual stressful incidents but rather on the coping style, which is assessed by I-E. In this study, the Kessler 6 (K6) scale was employed to assess the psychological distress of employees. Occupational safety and health (OSH) programs typically invite employees to complete a voluntary health assessment questionnaire consisting of brief self-report health scales (as opposed to a diagnosis). K6 is a brief, well-validated scale that assesses psychological distress and effectively predicts mental disorders [4, 5]. In 1998, the number of suicides in Japan increased sharply, particularly among middle-aged men (i.e., a productive age group). Consequently, Japan has one of the highest suicide rates among developed countries, presenting a significant problem for the country, and mental health problems are blamed for the majority of the reported suicides [5, 6].

The primary concern of this study is the possible effect of coping styles as assessed by the I-E on psychological distress in employees. The data presented in this paper identifies the sociodemographic and work-related factors that impact the relationship between coping styles and psychological distress. The results presented in this study can be easily incorporated by OSH professionals in future employee-health risk assessment surveys. Moreover, this information about the factors of psychological distress and coping styles may improve mental health clinicians' understanding of employees.

## 2. Materials and Methods

### 2.1. Participants

The information presented in this paper was collected as part of the Akita Occupational Health Promotion Center's Study for Mental Health [5]. This project involves conducting a study designed to investigate stressful situations and stress management skills and to assess psychological distress in employees. The participants in this study were recruited as follows. Randomly selected employers were recruited (random systematic sampling) and their employees were invited to complete the self-administered questionnaire. In all, fifteen employers from public and private sector firms in Akita prefecture, Japan, agreed to participate in the study. The questionnaires were distributed to the participants using paper-based methods. Employees in the present study were invited to answer the surveys for each company during a one-month survey period (September-October 2007). Participation in the survey was voluntary and confidential. In addition, this study obtained sociodemographic information from the participants. The demographic information collected during this study included the sex, age distribution (29 years or younger, 30 to 39 years, 40 to 49 years, and 50 years and older), and the highest level of education obtained. Across Japan, nine years of compulsory education, which includes elementary school and junior high school, is recognized as the minimum education level; the next highest level of education is typically considered the completion of three years of senior high school. Therefore, education was categorized into compulsory and senior high school, tertiary education, and graduate degree or higher. The questionnaire survey also elicited information on the employees' occupational characteristics (i.e., full time, managerial class, and job category). They were asked to select their job category from the following possible choices: (1) clerical or administrative support (e.g., bookkeeper, administrative assistant, or office supervisor), (2) sales- or service-related occupation (e.g., sales representative, stockbroker, or retail sales staff), (3) professional or technical support (e.g., engineer, doctor, nurse, laboratory technician, or computer programmer), and (4) others (e.g., on-site worker, crafts worker, mechanic, or driver). The present study also posed a question on the employees' average number of working hours per day. The Japan Labour Health and Welfare Organization, which has established occupational health promotion centers in each administrative division, approved the study protocol.

### 2.2. Instruments

 In this study, the I-E scale was used to measure coping style factors. Certain major problems have been reported in the use of Rotter's I-E scale1. First, the scores on Rotter's I-E scale have shown a consistent and significant relationship with social desirability [7, 8]. In addition, its face validity is low; as a number of authors have noted, Rotter's scale confounds personal, social, political, and ideological causation [9–11]. In response to these problems, Kambara and his colleagues developed an alternative measure of I-E [12, 13]. They named their 18-item scale (9 items each for internality and externality) the “Japanese version of the locus of control scale.” Each item is evaluated using a 4-point rating scale ranging from “value = 1” to “value = 4.” The respondent is instructed to indicate a degree of agreement or disagreement with each item on the 4-point scale. Therefore, the sum of the response scores can range from 18 to 72—high scores indicate internality. Further analyses employing this scale can be found in Kambara et al. (2001) [12]. In the previous report, internal consistency reliability was estimated at 0.78, and the test-retest reliability was 0.76 [12, 13]. In the current study, this scale was used instead of the original I-E scale.

As mentioned above, the current study also administered the K6 scale (30-day prevalence) to assess psychological distress. Each of the six items on K6 is rated on a five-point scale ranging from “none of the time” (value = 0) to “all the time” (value = 4). Therefore, the sum of the response scores can range from 0 to 24. The psychological dimensions explored in K6 make it sensitive and specific to mental disorders like affective and anxiety disorders [4, 5].

### 2.3. Analytical Procedure

Statistical analyses on cross-tabulations of the I-E scores, K6 scores, and sociodemographic and employment-related variables were performed using SPSS version 11.0J for Windows (SPSS, Tokyo, Japan). Statistical differences for cross-tabulations in the sex and age distribution of each category were analyzed using Pearson's *χ* ^2^ statistic. Furthermore, the Mann-Whitney *U* test was used to measure the statistical differences in sex and age distribution with regard to the values of the I-E and K6 scores. The correlations between the I-E and K6 scales in sex and age distribution were analyzed using Spearman's rank correlation. Furthermore, stepwise multiple regression analyses were performed to assess the effects of related factors, and three regression analyses—with the dependent variable as the I-E score—were performed. In one regression, sex was included as an independent variable. The remaining two regressions were conducted on separate data sets for males and females.

## 3. Results

Of the 2,145 employees, 1,873 responded to the questionnaire (response rate: 87.3%); however, the number of questionnaires with satisfactory responses, excluding those with insufficient data, was 1,533 (71.5%), which included 632 males and 901 females. The respective Cronbach's alphas for “internality” and “externality” in the I-E scale were 0.77 and 0.72; Cronbach's alpha for the K6 scale was 0.88.  Table 1 divides the participants according to their sex and summarizes the information pertaining to sociodemographic status and employment-related variables. With regard to the differences between males and females, Pearson's *χ* ^2^ test revealed significant differences (*P* < 0.001) in age, education, employee type (managerial or nonmanagerial class), job category, and number of working hours per day. However, there was no significant difference in employment status (full-time or part-time work).

Table 2 presents the mean and standard deviations of the scores of the I-E scale on the basis of sex and age distribution. With regard to the sex-based differences, significant differences were observed solely in the 40–49 years age group (*P* < 0.05; Mann-Whitney *U* test). Other age groups showed no significant sex-based differences.  Table 3 presents the mean and standard deviations of the scores of the K6 scale on the basis of sex and age distribution. For all age groups except the 30–39 years group, the mean K6 scores of females were higher than those of males. Furthermore, the older age groups tended to have lower K6 scores with the exception of the males in this age group. Significant sex-based differences were observed in the 29 years or younger age group (*P* < 0.05), 50 years and older (*P* < 0.01), and all the age groups (*P* < 0.01; Mann-Whitney *U* test).  Table 4 presents the correlation between the I-E and K6 scales subdivided by sex and age distributions. Both the males and females in all the age groups showed significantly negative correlations (*P* < 0.01; Spearman's rank correlation).  Table 5 presents the effects of psychological distress (K6 scale), sociodemographic, and employment-related factors on the I-E scale using multiple regression analyses; unstandardized (B) and standardized (*β*) regression coefficients are provided. The analysis of the effects of K6 scores, sociodemographic status, and employment-related variables on the I-E scores indicates that the independent effects of sex (*P* < 0.05), compulsory/senior high school education (*P* < 0.01), managerial employee type (*P* < 0.05), and K6 score (*P* < 0.01) on the I-E score were significant for all participants. Furthermore, only the K6 score was significant for males (*P* < 0.01). Conversely, for females, the 40–49 years age group (*P* < 0.01), 50 years and older age group (*P* < 0.05), compulsory/senior high school education (*P* < 0.01), full-time employment status (*P* < 0.01), managerial employee type (*P* < 0.05), and K6 score (*P* < 0.01) were significant. The effects of the variable of number of working hours per day were insignificant in all three sets. Therefore, the K6 score was the only score that was significant for all three sets.

## 4. Discussion

The response rate of 87.3% obtained by this survey was much higher than the typical response rate obtained in the case of employee-administered health questionnaires in many large organizations [5, 14]. The I-E was hypothesized to moderate stressor-strain relations because it appears as the factor most likely to affect the coping styles of individuals. Comparatively little research has been conducted on how coping styles interact with job-related psychological distress assessed by a psychological distress scale in an applied setting, although factors related to coping styles such as I-E have a lengthy tradition of research. Therefore, the discussion first addresses the principal concern of the study, which is the relationship between the effects of coping styles (I-E) and psychological distress (K6). Following this, some consideration is given to the effects that related sociodemographic and occupational factors have on these variables. On observing the direct correlations between I-E and K6 (Spearman's rank correlation), a uniform pattern of findings emerged—all the significant correlations were negative, indicating that, as the I-E score increased (greater internality), the K6 score decreased (less psychological distress). These results are in accordance with those observed in earlier research on psychological distress from job-related stressors, such as job demands [15, 16]. For instance, externals are likely to undergo greater psychological distress than others. In contrast, internals are likely to undergo less psychological distress, even if they have relatively many stressors. The simplest explanation for the observation that externals report both greater job-related stressors and psychological distress is that they perceive themselves as being more environment dependent, with their life rewards more likely to be viewed as a matter of fate, chance, or luck [1, 17]. However, the moderator results suggest that the picture is more complicated than this since I-E interacts with specific job-related stressors in its relationship with psychological distress (i.e., the effects of I-E on the stressor-strain relationship differ according to the subtype of job-related stressors such as role conflict, role ambiguity, qualitative role low-load, quantitative role high-load, and environmental frustration [3]). Some previous studies report the relationships between I-E and psychological distress from these subtypes of stressors [3, 18–21]. Moreover, some reports add the factor of Type A to the relationship between I-E and psychological distress. It has been generally reported that persons with Type A personalities have increased psychological distress, as do persons with high external LOC. Furthermore, persons with Type A personalities and external LOC undergo greater psychological distress than those with Type A personalities and internal LOC, that is, Type A persons are generally likely to have greater psychological distress, which is exacerbated if they are externals [22]. In addition, low levels of hardiness, self-respect, tendency of avoidance-oriented coping behavior, and external LOC are related to burnout [23]. Consequently, externals are more likely to experience burnout. Furthermore, persons with low levels of self-confidence and self-esteem and persons with a lack of self-efficacy (i.e., externals) are likely to adopt non-adoptive coping behaviors such as avoidance-oriented coping behaviors. Such persons are more likely to have increased burnout.

Other variables that moderate the stressor-strain relationship are sex and age. For example, male teachers reported greater burnout and lower job satisfaction than that among female teachers. In addition, although male department heads scored significantly higher on psychological burnout, there were no sex differences in the measures of satisfaction and emotional well-being [24]. Another report shows that age is related to personal accomplishment and professional commitment but inversely related to emotional exhaustion, that is, younger subjects are likely to have a higher level of burnout [25]. Varieties of studies and results demonstrate the effect of behavioral control, such as I-E, on psychological distress. In sum, they remain possible conjectures from the present findings and provide interesting possibilities for future research.

In this study, the K6 scale was used to evaluate the degree of psychological distress. The psychological dimensions explored in K6 make it sensitive and specific to mental disorders such as affective and anxiety disorders [4]. It is reasonable to assume that the severity of mental health symptoms (degree of psychological distress) is primarily responsible for reduced performance at work [5, 26, 27]. K6 is one of the most widely used psychological distress scales across the globe. Therefore, it is ideal for inclusion in health risk assessment for OSH. However, there is a dearth of published large-scale, normative values that specifically pertain to the workforce. This study is one of the few reports where employees' psychological distress is assessed using K6 by varying the demographic, employment-related, and individual personality variables.

A limitation of this study was cross-sectional sampling, which made it difficult to infer causality. The data sample was selected at random, but companies (employers) decided to participate in the project, and the employees from these companies decided to respond to the survey. However, self-selection biases in the current data are representative of those inherent in typical employee health assessment surveys. Moreover, the structured interview method is not feasible in large sample studies (as in this case), so some alternative method must be employed. The assessment of factor-related coping with stressors is another limitation of this study because it was based on a single questionnaire measurement (I-E). As previously noted, one reason for studying long-term coping styles is that not all coping is synchronous with stressful incidents or episodes; psychological distress builds up over months or years rather than being the response to a single stressful incident. One approach to investigating the long-term patterns of coping styles is to measure the coping behavior repeatedly. However, in this type of research design, the response obtained may in part be an artifact of the method utilized by repeatedly focusing their attention on how they cope in the long term [3]. An alternative to this approach, which reduces the occurrence of such problems, is to examine the extent to which job stressor-strain relations are accentuated by certain coping styles. Since long-term patterns of coping styles are being examined, the necessity of performing frequent repeated measures is reduced.

## 5. Conclusions

This study reveals the relationship between the I-E scale as an indicator of coping styles and the K6 scale as an indicator of psychological distress and the effects of sociodemographic and employment-related factors on this relationship. The effect of the K6 scale was significant for the I-E scale for all three sets (all participants, males, and females). Further research should be conducted on the relationship between the factors related to coping style and the psychological distress scale since there are only a few studies directly examining this relationship. The information obtained by this study related to the factors of psychological distress and its coping style will hopefully improve mental health clinicians' understanding of employees.

## Figures and Tables

**Table 1:** Sociodemographics of the sample and the differences between the males and females.

	All participants		Males		Females	
	*N*	(%)	*N*	(%)	*N*	(%)
Total	1533	(100)	632	(100)	901	(100)
Age*						
≤29	337	(22.0)	116	(18.4)	221	(24.5)
30–39	415	(27.1)	154	(24.4)	261	(29.0)
40–49	402	(26.2)	153	(24.2)	249	(27.6)
≥50	379	(24.7)	209	(33.1)	170	(18.9)

Education*						
Compulsory/senior high school	708	(46.2)	431	(68.2)	277	(30.7)
Some tertiary education	706	(46.1)	127	(20.1)	579	(64.3)
Graduate degree or higher	99	(6.5)	63	(10.0)	36	(4.0)
Unknown	20	(1.3)	11	(1.7)	9	(1.0)

Employment status^†^						
Full-time work	1361	(88.8)	569	(90.0)	792	(87.9)
Part-time work	160	(10.4)	59	(9.3)	101	(11.2)
Unknown	12	(0.8)	4	(0.6)	8	(0.9)

Employee type*						
Managerial class	165	(10.8)	112	(17.7)	53	(5.9)
Nonmanagerial class	1346	(87.8)	517	(81.8)	829	(92.0)
Unknown	22	(1.4)	3	(0.5)	19	(2.1)

Job category*						
Clerical/administrative	209	(13.6)	111	(17.6)	98	(10.9)
Sales/service	172	(11.2)	101	(16.0)	71	(7.9)
Professional/technical	784	(51.1)	216	(34.2)	568	(63.0)
Others (on-site workers, etc.)	318	(20.7)	179	(28.3)	139	(15.4)
Unknown	50	(3.3)	25	(4.0)	25	(2.8)

Working hours per day*						
8 hours or less	829	(54.1)	260	(41.1)	569	(63.2)
More than 8 hours	693	(45.2)	369	(58.4)	324	(36.0)
Unknown	11	(0.7)	3	(0.5)	8	(0.9)

Significances representing the differences between males and females (Pearson's *χ* ^2^ statistic).

*P < 0.001, ^†^not significant.

**Table 2:** Scores of internality-externality (locus of control) scale by sex and age distribution.

Age	All participants	Males	Females
	Mean ± SD	Mean ± SD	Mean ± SD
≤29^†^	47.37 ± 6.80	47.62 ± 7.42	47.24 ± 6.46
30−39^†^	47.43 ± 6.62	47.31 ± 7.57	47.50 ± 6.00
40−49*	46.87 ± 6.99	47.87 ± 7.90	46.26 ± 6.30
≥50^†^	47.25 ± 7.10	47.81 ± 6.72	46.55 ± 7.51

Total^†^	47.22 ± 6.87	47.67 ± 7.34	46.91 ± 6.51

Significance scores representing the differences between males and females (Mann-Whitney *U* test).

*P < 0.05, ^†^not significant.

**Table 3:** Scores of Kessler 6 (K6) psychological distress scale by sex and age distribution.

Age	All participants	Males	Females
	Mean ± SD	Mean ± SD	Mean ± SD
≤29*	6.58 ± 5.24	5.84 ± 5.37	6.97 ± 5.13
30–39^†^	6.26 ± 5.23	6.39 ± 5.56	6.18 ± 5.04
40–49^†^	5.91 ± 4.96	5.77 ± 5.08	6.00 ± 4.90
≥50**	4.93 ± 4.45	4.41 ± 4.25	5.58 ± 4.62

Total**	5.91 ± 5.01	5.48 ± 5.06	6.21 ± 4.96

Significance scores representing the differences between males and females (Mann-Whitney *U* test).

*P < 0.05, **P < 0.01, ^†^not significant.

**Table 4:** Correlation between the internality-externality (locus of control) scale and Kessler 6 (K6) scale by sex and age distribution (Spearman's rank correlation).

Age	All participants	Males	Females
	*r*_*s*_	*r*_*s*_	*r*_*s*_
≤29	−0.379*	−0.273*	−0.443*
30–39	−0.331*	−0.407*	−0.284*
40–49	−0.315*	−0.348*	−0.287*
≥50	−0.310*	−0.348*	−0.253*

Total	−0.328*	−0.344*	−0.311*

*r*_*s*_: Spearman's rank correlation coefficient, *P < 0.01.

**Table 5:** Effects of psychological distress, sociodemographic, and employment-related factors on the internality-externality (locus of control) scale (stepwise multiple regression analysis).

	Extracted factors	B	*β*	
	Sex	−0.847	−0.061	*
All participants	Compulsory/senior high school (Education)	−1.471	−0.107	**
(*R* ^2^ = 0.146)	Managerial class (Employee type)	−1.204	−0.055	*
	Kessler 6 (K6) score	−0.500	−0.365	**

Male	Kessler 6 (K6) score	−0.570	−0.391	**
(*R* ^2^ = 0.153)				

	Age 40–49	−1.379	−0.095	**
	Age 50-	−1.362	−0.082	*
Female	Compulsory/senior high school (Education)	−2.235	−0.159	**
(*R* ^2^ = 0.156)	Full-time working (Employment status)	1.994	0.097	**
	Managerial class (Employee type)	−1.793	−0.066	*
	Kessler 6 (K6) score	−0.466	−0.357	**

*R* ^2^: coefficient of determinant; B: regression coefficient; *β*: standardized regression coefficient.

*P < 0.05, **P < 0.01.
